# Optimizing heterologous protein production in the periplasm of *E. coli* by regulating gene expression levels

**DOI:** 10.1186/1475-2859-12-24

**Published:** 2013-03-12

**Authors:** Susan Schlegel, Edurne Rujas, Anders Jimmy Ytterberg, Roman A Zubarev, Joen Luirink, Jan-Willem de Gier

**Affiliations:** 1 Center for Biomembrane Research, Department of Biochemistry and Biophysics, Stockholm University, Stockholm SE-106 91, Sweden; 2 Chemistry I Division, Department of Medical Biochemistry and Biophysics, Karolinska Institute, Stockholm, Sweden; 3 Section of Molecular Microbiology, Department of Molecular Cell Biology, VU University, De Boelelaan 1085, Amsterdam, 1081 HV, The Netherlands

**Keywords:** Recombinant protein, Protein production, *Escherichia coli*, Lemo21(DE3), Protein translocation, Periplasm, Sec-translocon

## Abstract

**Background:**

In *Escherichia coli* many heterologous proteins are produced in the periplasm. To direct these proteins to the periplasm, they are equipped with an N-terminal signal sequence so that they can traverse the cytoplasmic membrane *via* the protein-conducting Sec-translocon. For poorly understood reasons, the production of heterologous secretory proteins is often toxic to the cell thereby limiting yields. To gain insight into the mechanism(s) that underlie this toxicity we produced two secretory heterologous proteins, super folder green fluorescent protein and a single-chain variable antibody fragment, in the Lemo21(DE3) strain. In this strain, the expression intensity of the gene encoding the target protein can be precisely controlled.

**Results:**

Both SFGFP and the single-chain variable antibody fragment were equipped with a DsbA-derived signal sequence. Producing these proteins following different gene expression levels in Lemo21(DE3) allowed us to identify the optimal expression level for each target gene. Too high gene expression levels resulted in saturation of the Sec-translocon capacity as shown by hampered translocation of endogenous secretory proteins and a protein misfolding/aggregation problem in the cytoplasm. At the optimal gene expression levels, the negative effects of the production of the heterologous secretory proteins were minimized and yields in the periplasm were optimized.

**Conclusions:**

Saturating the Sec-translocon capacity can be a major bottleneck hampering heterologous protein production in the periplasm. This bottleneck can be alleviated by harmonizing expression levels of the genes encoding the heterologous secretory proteins with the Sec-translocon capacity. Mechanistic insight into the production of proteins in the periplasm is key to optimizing yields in this compartment.

## Background

*Escherichia coli* is the most widely used bacterial vehicle to produce heterologous proteins [1]. Proteins are increasingly produced in the periplasm [2-5]. It is easier to isolate proteins from this compartment than from whole cell lysates, and, more importantly, in the oxidizing environment of the periplasm the disulfide bond formation (Dsb)-system catalyzes the formation of disulfide bonds. Therefore, disulfide bond containing proteins, like antibody fragments and many peptide hormones, are produced in the periplasm to enable folding into their native conformation [4,6].

**Figure 1:**
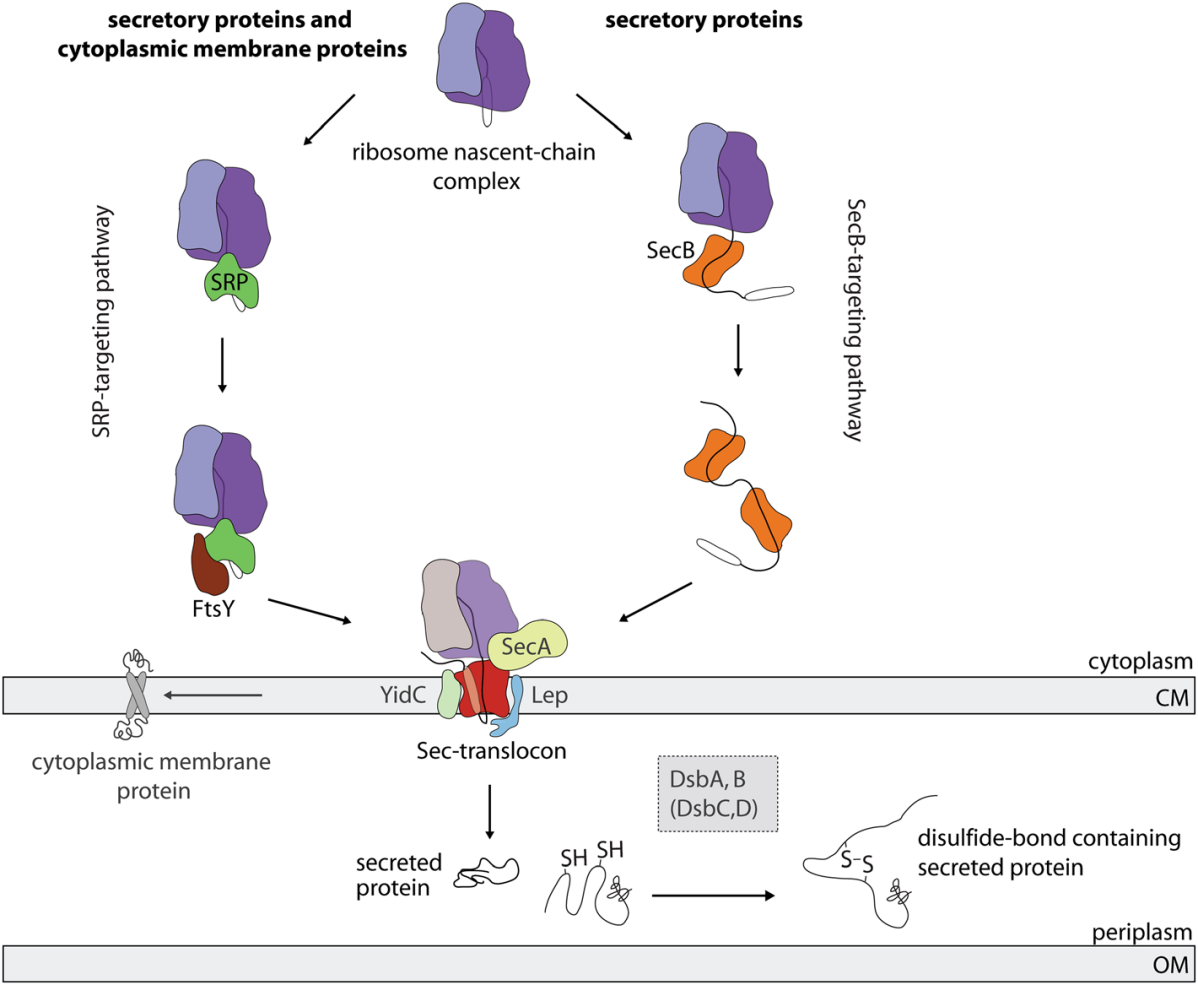
**The biogenesis of Sec-translocon dependent secretory and cytoplasmic membrane proteins in *E. coli*. **In *E. coli*, most secretory and cytoplasmic membrane proteins require the Sec-translocon for their biogenesis. The Sec-translocon is a protein conducting channel in the cytoplasmic membrane (CM), which mediates the vectorial transfer of secretory proteins across and the biogenesis of membrane proteins in the cytoplasmic membrane [7]. Secretory proteins are equipped with a cleavable N-terminal signal sequence. The signal sequence determines whether a secretory protein is targeted to the Sec-translocon *via *the post-translational SecB-targeting pathway or the co-translational signal recognition particle (SRP)-targeting pathway, which is comprised of the SRP and its receptor FtsY. Upon translocation, the signal sequence is cleaved off by leader peptidase (Lep) and the secretory protein is released into the periplasm. In this compartment, the Dsb-system can catalyze the formation of disulfide bonds. The disulfide oxidoreductase DsbA catalyzes the *de-novo *formation of disulfide bonds in polypeptide chains. The disulfide bond formation protein B (DsbB) is essential to maintain DsbA in an oxidized state. Incorrectly formed disulfide bonds can be corrected by DsbC/D. For a more detailed description of disulfide bond formation in the periplasm of *E. coli *see [3,5]. Cytoplasmic membrane proteins are targeted to the Sec-translocon *via* the SRP-targeting pathway. SecA = peripheral membrane ATPase associated with the Sec-translocon [18], OM = outer membrane, YidC = cytoplasmic membrane protein translocase/insertase [18].

In order to reach the periplasm, the heterologous proteins are equipped with an N-terminal signal sequence that guides them to the Sec-translocon, which is a protein-conducting channel in the cytoplasmic membrane [7]. Two pathways can guide proteins to the Sec-translocon, the post-translational SecB-targeting pathway and the co-translational signal recognition particle (SRP)-targeting pathway [8] (Figure 1). The nature of the signal sequence is decisive for the choice of the targeting pathway [9-11]. The relatively hydrophobic DsbA signal sequence, which directs proteins to the Sec-translocon in an SRP-dependent fashion, is a widely used signal sequence for the production of heterologous secretory proteins [12-15]. The Sec-translocon mediates the vectorial transfer of secretory proteins across the cytoplasmic membrane. Subsequently, the signal sequence is clipped off by leader peptidase [7,16]. In the periplasm, the Dsb-system mediates the formation of disulfide bonds and various catalysts guide the folding process [5,17] (Figure 1).

To obtain high yields of a recombinant protein, the gene encoding this protein is usually expressed at the highest level possible. Unfortunately, the production of proteins that carry a signal sequence is, for yet poorly understood reasons, often toxic to the cell [6]. This negatively affects their yields in the periplasm. As has been observed for secretory proteins, the production of membrane proteins is also often toxic to *E. coli* and, as a consequence, yields are low. In this bacterium, most cytoplasmic membrane proteins are targeted to the Sec-translocon in a co-translational fashion *via* the SRP-targeting pathway [18] (Figure 1). Recently, we have shown that the saturation of the Sec-translocon capacity is the main bottleneck in the production and localization of membrane proteins in the cytoplasmic membrane in *E. coli*[19-21]. The Lemo21(DE3) strain was critical to further our understanding of the effect of the saturation of the Sec-translocon capacity as a major bottleneck in membrane protein production [20,21]. In this strain, the expression levels of the gene of interest, i.e., the number of transcripts synthesized, can be precisely controlled over a wide range. Adjustment of the expression level of the gene encoding the membrane protein of interest such that the Sec-translocon capacity is no longer saturated can minimize the toxic effects of membrane protein production and protein levels in the membrane can be optimized [20,21].

Here, we present how cell physiology and periplasmic protein production are affected by varying gene expression levels using two heterologous proteins, super folder green fluorescent protein (SFGFP) [22,23] and a single-chain variable antibody fragment (scFv) in Lemo21(DE3). Both proteins were equipped with a DsbA-derived signal sequence. This approach enabled us to identify the Sec-translocon capacity as a major bottleneck hampering the periplasmic production of heterologous proteins. By harmonizing gene expression levels with the capacity of the Sec-translocon, protein production in the periplasm can be optimized.

## Results

### Modulating gene expression levels using Lemo21(DE3)

**Figure 2:**
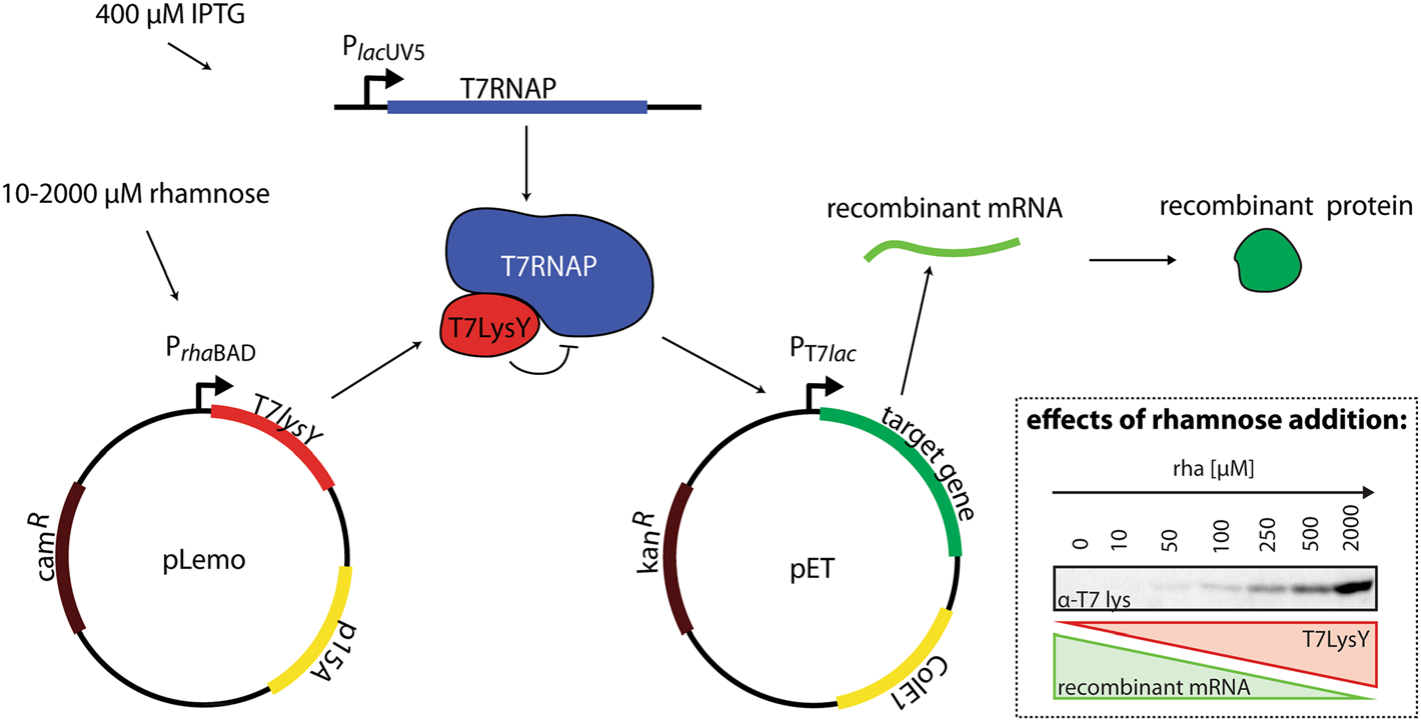
**Regulating target gene expression levels using the Lemo21(DE3) strain.** Lemo21(DE3) is a BL21(DE3) derivative harboring the pLemo plasmid. In Lemo21(DE3), expression of the gene encoding the target protein is driven by T7 RNAP. The gene encoding T7 RNAP is located on the chromosome. Its expression is governed by the not well-titratable and very strong, IPTG inducible *lac*UV5 promoter. The activity of T7 RNAP can be modulated by expression of the gene encoding the natural inhibitor of the T7 RNAP, T7 lysozyme, from pLemo. The pLemo plasmid has a p15A ori and a chloramphenicol resistance marker. Expression of the gene encoding the T7 lysozyme is governed by the well-titratable rhamnose promoter. The gene encoding the target protein is located on a pET-vector. pET-vectors have a ColE1 ori and the version used in this study has a kanamycin resistance marker. The expression of the gene encoding the target protein from the pET-vector is governed by the T7*lac *promoter. The expression levels of the gene encoding the target protein can be increasingly dampened by the addition of increasing amounts of rhamnose to the culture. The more rhamnose is added the more T7 lysozyme is synthesized (see immuno-blot of T7 lysozyme on the right). As a consequence, T7 RNAP is increasingly inhibited and the expression levels of the target gene decrease (see inset).

To study the effects of varying expression levels of genes encoding heterologous, secretory proteins on the physiology of the cell and the yields of the proteins in the periplasm, we used two heterologous proteins, SFGFP and the scFv BL1 [22-25]. SFGFP folds unassisted and its fluorescent properties allow rapid detection. The scFv BL1 specifically recognizes *E. coli* β-galactosidase, providing an easy diagnostic tool for folding and activity. A DsbA-derived signal sequence was used to direct these two proteins to the Sec-translocon. To set varying gene expression levels, both secretory proteins were produced in Lemo21(DE3) (Figure 2). Lemo21(DE3) is derived from BL21(DE3) [20]. In BL21(DE3) expression of the target gene is driven by T7 RNA polymerase (RNAP). Expression of the gene encoding T7 RNAP is controlled by the isopropyl-β-D-thiogalactoside (IPTG) inducible *lac*UV5 promoter, which is poorly titratable [26]. To create Lemo21(DE3), BL21(DE3) was transformed with the pLemo plasmid, which harbors the gene encoding the T7 RNAP inhibitor T7 lysozyme [20]. The expression of this gene is governed by the well-titratable rhamnose promoter [27] (Figure 2, immuno-blot right panel inset). When the gene of interest is cloned in a T7 promoter based expression vector, Lemo21(DE3) allows screening a wide window of expression levels of the target gene by adding varying amounts of rhamnose.

### Expression levels of the gene encoding secretory SFGFP affect its accumulation levels in the periplasm

**Figure 3:**
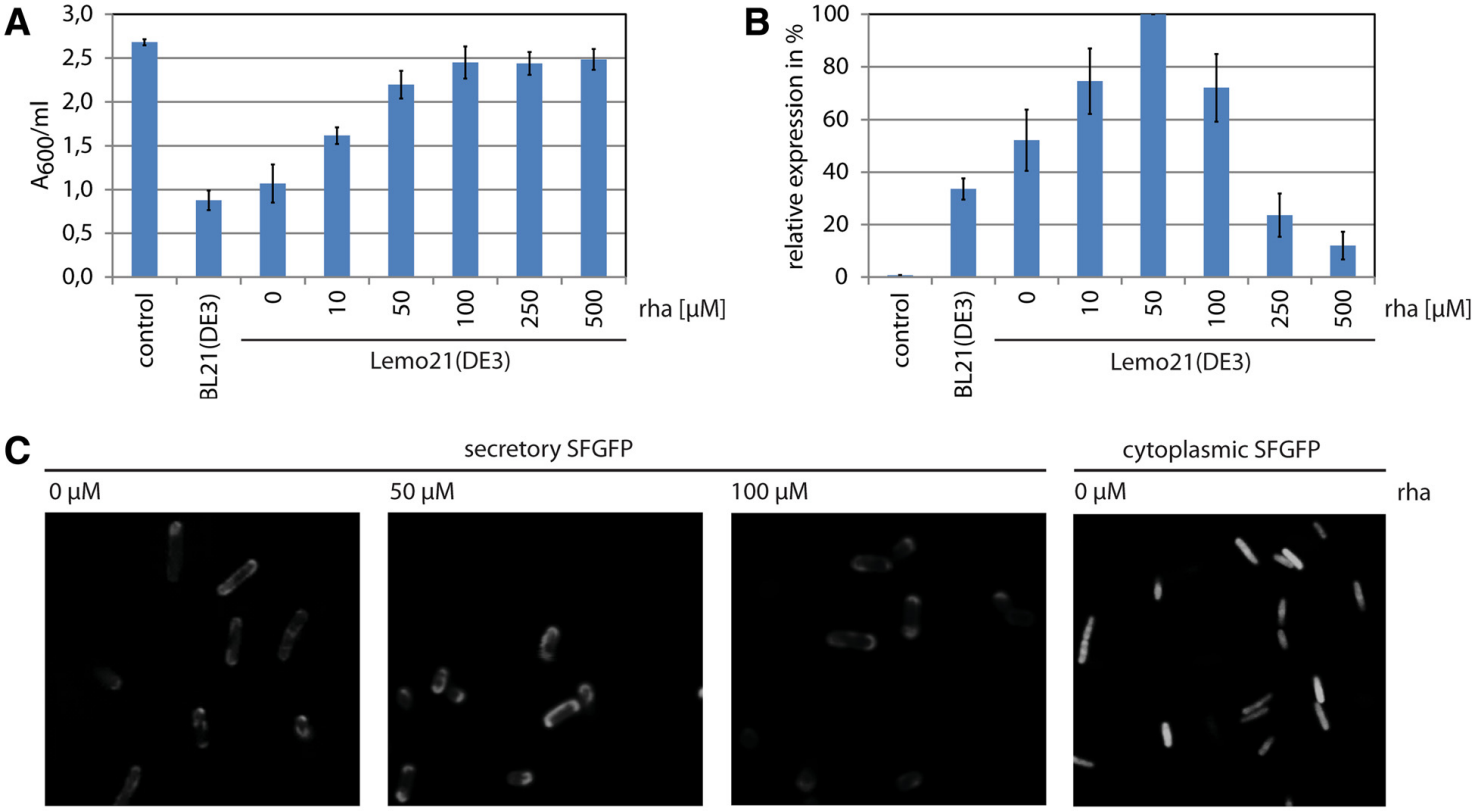
**Production of secretory SFGFP following varying gene expression levels.** Lemo21(DE3) cells harboring a pET-vector with the gene encoding secretory SFGFP were cultured in LB medium at 30°C. The expression of secretory SFGFP was induced with 400 μM IPTG for 4 h. Rhamnose was present as indicated. Lemo21(DE3) harboring an empty expression vector (control) and BL21(DE3) producing secretory SFGFP were included as controls. **A **The effect of the production of secretory SFGFP following varying gene expression levels on biomass formation was monitored by measuring the A_600_. **B **The effect of the production of secretory SFGFP following varying gene expression levels on protein yields was monitored as fluorescence per ml of culture. The highest level of fluorescence was set to 100%; the other values were adjusted accordingly. **C **The localization of secretory SFGFP in Lemo21(DE3) cells cultured in the absence and presence of increasing concentrations of rhamnose was monitored directly in whole cells using fluorescence microscopy. Lemo21(DE3) cells producing cytoplasmic SFGFP (i.e., SFGFP not equipped with a signal sequence) in the absence of rhamnose were included as control.

In *E. coli*, SFGFP is the only known GFP variant that can fold into its fluorescent form upon translocation *via* the Sec-translocon, making it an ideal first target [22,28]. The gene encoding secretory SFGFP was expressed from a T7 promoter based expression vector in Lemo21(DE3) cultured in the absence and presence of increasing amounts of rhamnose (Figure 3). Throughout, BL21(DE3) harboring the expression vector with the gene encoding secretory SFGFP and Lemo21(DE3) harboring an empty expression vector were used as references.

Culturing Lemo21(DE3) cells in the presence of increasing amounts of rhamnose resulted in an increase in biomass formation as determined by A_600_ measurements (Figure 3A). SFGFP production was monitored by whole cell fluorescence measurements (Figure 3B). Whole cell fluorescence (fluorescence per ml) peaked at a rhamnose concentration of 50 μM. Fluorescence microscopy revealed a halo of fluorescence (Figure 3C). To determine if this halo originated from soluble SFGFP, cells were broken and separated into a soluble and a non-soluble fraction. The fluorescent signal originating from the halo was detectable in the soluble fraction (results not shown). The production of SFGFP without a signal sequence in Lemo21(DE3), led to the cytoplasm of the cells being fluorescent (Figure 3C). This indicates that the secretory SFGFP is directed to the periplasm. For more detailed information concerning the localization of SFGFP in *E. coli* see the comment in the Additional file 1. Expression of the gene encoding secretory SFGFP in the BL21(DE3) strain under standard conditions (see Methods) corresponded to the expression in Lemo21(DE3) in the absence of rhamnose (Figure 3A, B). Clearly, BL21(DE3) is far from optimal for the production of SFGFP in the periplasm.

### Consequences of the production of SFGFP following varying gene expression levels

**Figure 4:**
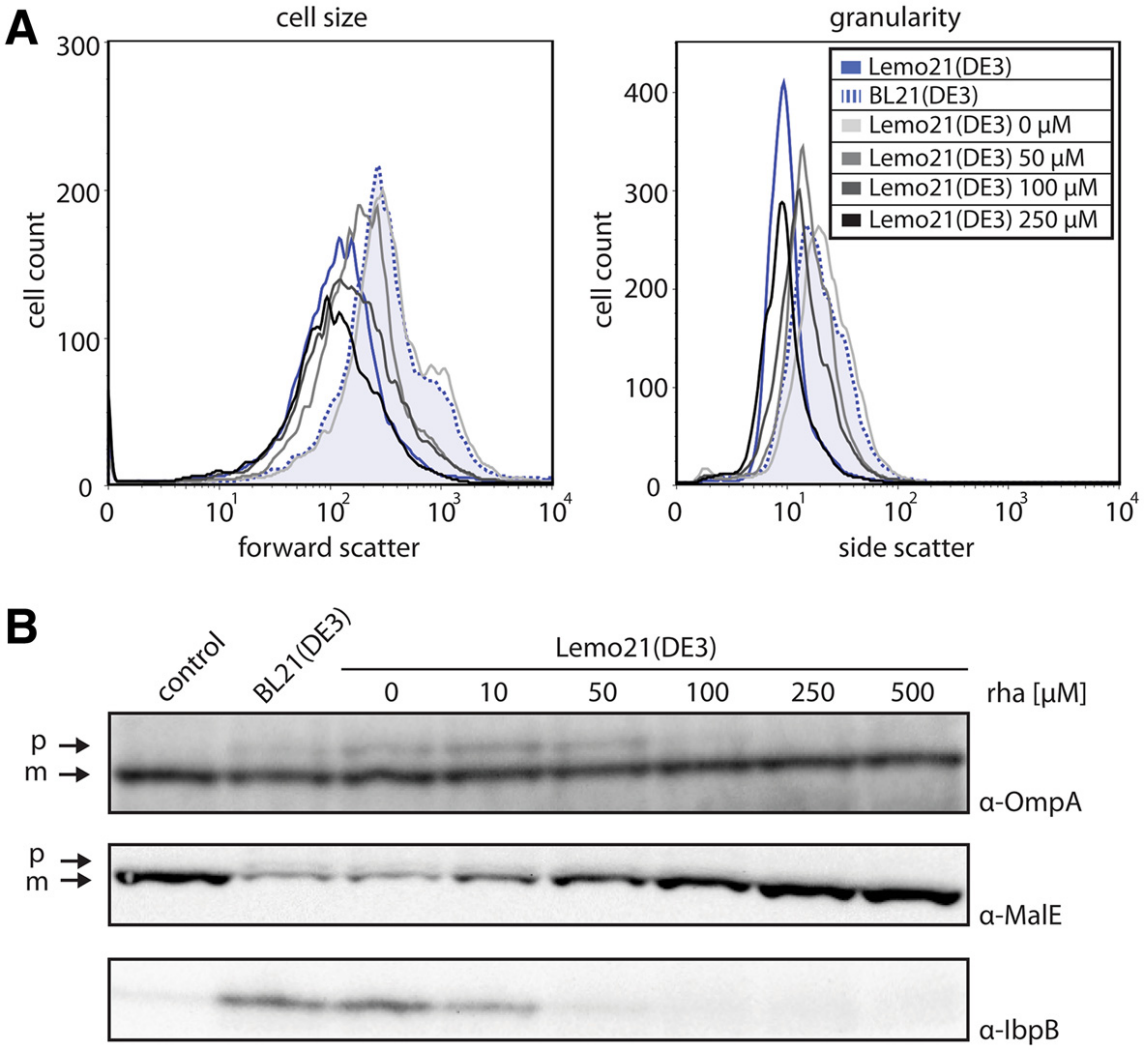
**Consequences of varying expression levels of the gene encoding secretory SFGFP.** The expression of the gene encoding secretory SFGFP was induced with IPTG in Lemo21(DE3) in the absence and presence of increasing concentrations of rhamnose. Lemo21(DE3) harboring an empty expression vector (control) and BL21(DE3) harboring the secretory SFGFP expression vector were included as controls. 4 h after induction of the expression of the gene encoding secretory SFGFP cells were analysed by flow cytometry and immuno-blotting. **A **Cell size (forward scatter) and granularity (side scatter) were monitored in cells producing secretory SFGFP. **B **SDS-PAGE/immuno-blotting using antisera against IbpB, OmpA and MalE. For the immuno-blotting analysis, the precursor (p) and the mature form (m) of OmpA and MalE are indicated.

Our data show that decreasing expression levels of the gene encoding secretory SFGFP led to a decrease of the observed negative (toxic) effect on biomass formation (Figure 3). To further our understanding of the observed effects we used a combination of flow cytometry and immuno-blotting (Figure 4).

Flow cytometry measurements indicated that both the cell size (forward scatter) and granularity (side scatter) decreased with decreasing expression levels of the gene encoding secretory SFGFP (i.e., increasing rhamnose concentrations) (Figure 4A). The decrease in cell size points to a gradual decrease in cell division defects whereas the decrease in granularity suggests diminished accumulation of inclusion bodies/aggregates.

To examine potential protein misfolding/aggregation in the cytoplasm, levels of inclusion body protein B (IbpB) were monitored using immuno-blotting (Figure 4B). The expression of the gene encoding IbpB is induced upon protein misfolding/aggregation in the cytoplasm [29]. At lower rhamnose concentrations cells contained significant levels of IbpB. In keeping with the results from the flow cytometry measurements, levels of IbpB decreased with an increase in rhamnose concentration. This suggests that there is a protein accumulation/folding problem in the cytoplasm if the expression level of the gene encoding a secretory protein is too high, which could be due to saturation of the Sec-translocon capacity.

To directly monitor if saturation of the Sec-translocon capacity indeed plays a role in the observed negative effects on biomass formation and protein homeostasis in the cytoplasm, the levels of the endogenous, SecB and Sec-translocon dependent secretory proteins, OmpA and MalE were determined using immuno-blotting [30-33] (Figure 4B). High expression level of the gene encoding secretory SFGFP in cells grown in the absence or presence of low rhamnose concentrations led to accumulation of precursor OmpA. For MalE, an increase in rhamnose concentration led to both a decrease in precursor MalE and an increase in the mature, secreted form of the protein. These findings corroborate that the toxicity observed at high gene expression levels stems from saturation of the Sec-translocon capacity.

It should be noted that, while visible, the precursor forms of OmpA and MalE did not accumulate in the cytoplasm to high levels. This is most likely due to their partial degradation.

### Effects of varying expression levels of the gene encoding a secretory scFv

To further explore the role of the Sec-translocon capacity in the periplasmic production of heterologous proteins, we used the scFv BL1. Notably, the version used in this study contains a C-terminal His-tag, facilitating its detection and purification.

**Figure 5:**
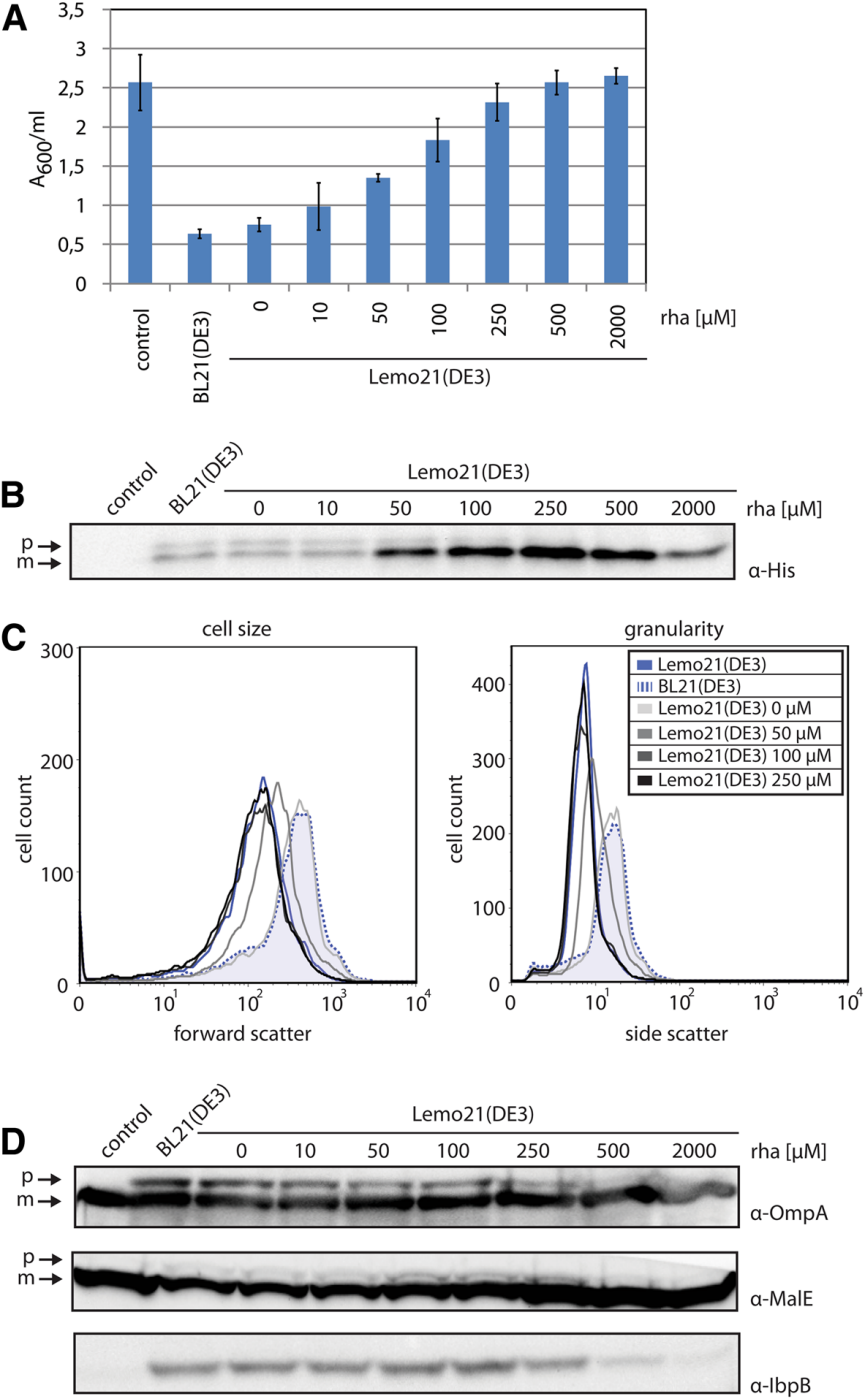
**Production of the secretory scFv BL1 following varying gene expression levels.** Expression of the gene encoding secretory BL1 was induced with IPTG in Lemo21(DE3) cells grown in the absence and presence of increasing concentrations of rhamnose. BL21(DE3) expressing the gene encoding secretory BL1 and Lemo21(DE3) harboring an empty expression vector were included as controls. **A **4 h after induction, cell growth was monitored by measuring A_600_. **B **Levels and processing of secretory BL1 were monitored using SDS-PAGE followed by immuno-blotting using an α-His antibody. The precursor (p) and the mature (m) form of the protein are indicated. **C **Using flow cytometry, cell size (forward scatter) and granularity (side scatter) of cells producing secretory BL1 were monitored. **D **Levels of IbpB, OmpA and MalE were monitored using a combination of SDS-PAGE and immuno-blotting. For OmpA and MalE, the precursor (p) and the mature form (m) of the respective protein are indicated.

First, the gene encoding secretory BL1 was expressed from a T7 promoter based expression vector in Lemo21(DE3) cultured in the absence and presence of increasing concentrations of rhamnose. BL21(DE3) harboring the expression vector with the gene encoding secretory BL1 and Lemo21(DE3) harboring an empty expression vector were used as references. As for secretory SFGFP, increasing concentrations of rhamnose (i.e., decreasing expression levels of the gene encoding secretory BL1) resulted in an increase in cell density (Figure 5A). Immuno-blotting using an antibody recognizing the C-terminal His-tag of BL1 showed that increasing amounts of rhamnose led to increasing amounts of processed, i.e., presumably periplasmically localized, BL1. Simultaneously, the non-processed, i.e., cytoplasmically localized, form decreased gradually and eventually disappeared (Figure 5B). Accumulation levels of the processed form of BL1 without any detectable precursor were highest at a rhamnose concentration of 500 μM. This concentration is apparently optimal for the production of the processed form of secretory BL1 only. Subcellular fractionation using the cytoplasmic chaperone GroEL, periplasmic chaperone SurA and the cytoplasmic T7 lysozyme as markers showed that, as expected, the processed form of BL1 was localized in the periplasm (Additional file 1: Figure S1) [34]. In addition, mass spectrometry was used to unambiguously show that BL1 produced in Lemo21(DE3) cells cultured at 500 μM rhamnose represents the processed form of the protein, i.e., BL1 lacking the signal sequence (Additional file 1: Figure S2). It should be noted that the levels of the precursor form of BL1 at sub-optimal rhamnose concentrations are relatively weak. This suggests that not properly targeted BL1 is partially degraded in the cytoplasm.

Subsequently, flow cytometry analysis showed that just as observed for secretory SFGFP, cell size and granularity decreased with increasing rhamnose concentrations (Figure 5C). The elevated IbpB levels in cells expressing secretory BL1 at sub-optimal rhamnose concentrations (Figure 5D) indicated that the observed granularity was due to the formation of inclusion bodies. In addition, immuno-blotting revealed the accumulation of precursor of endogenous secretory proteins (OmpA and MalE) and reduced levels of the mature form (MalE) at sub-optimal rhamnose concentrations (Figure 5D). All this points towards a folding/aggregation problem in the cytoplasm due to saturation of the Sec-translocon capacity.

Our observations indicate that the Sec-translocon capacity can be a major bottleneck when producing BL1 in the periplasm. Modulating the expression levels of the gene encoding secretory BL1 can be used to alleviate this bottleneck, thereby optimizing yields of processed BL1. It should be noted that optimal yields of processed BL1 could only be achieved within a narrow window of gene expression levels.

### Optimization of the expression level of the gene encoding secretory BL1 leads to functional protein in the periplasm

**Figure 6:**
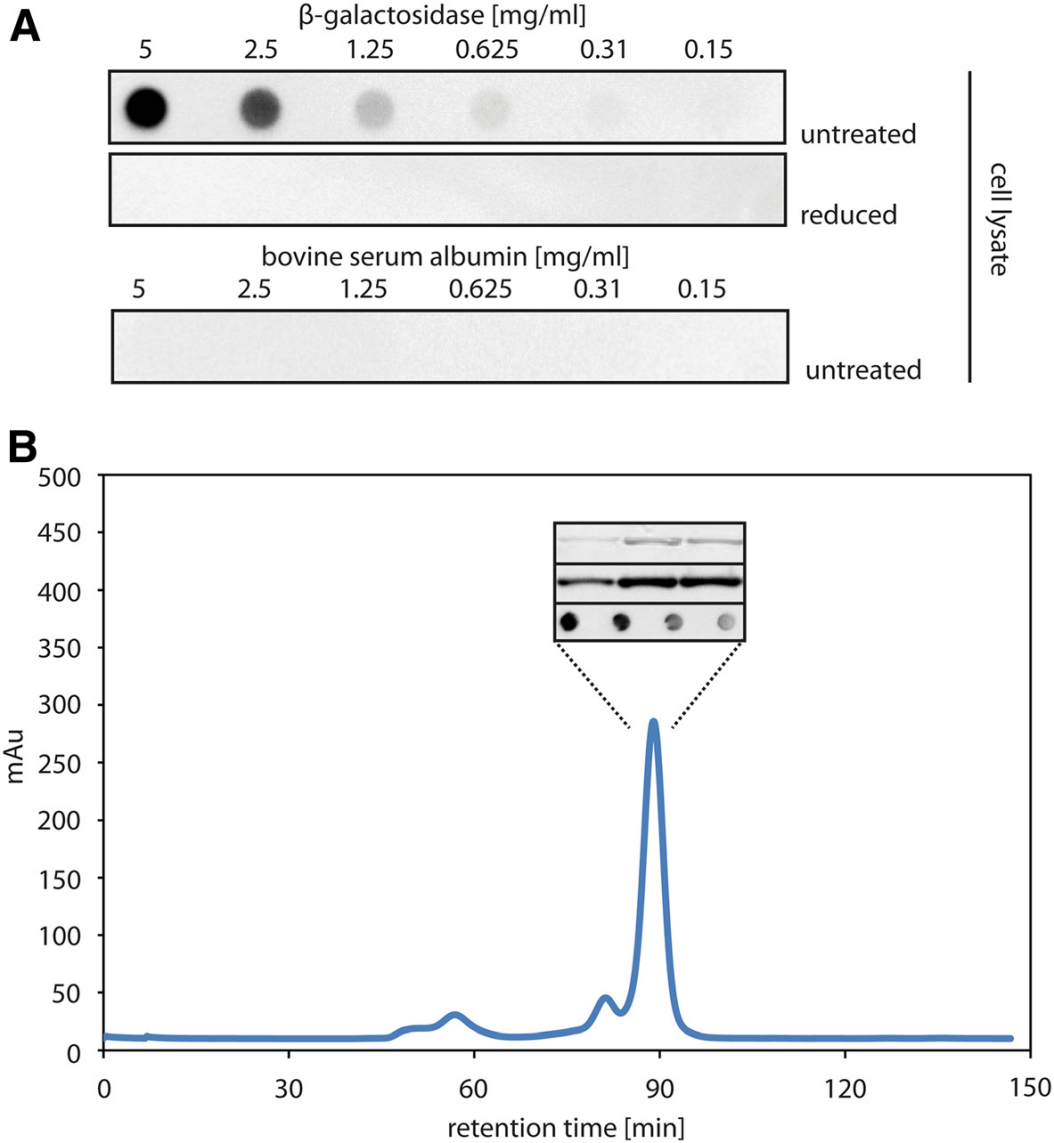
**Characterization of secretory BL1 expressed at the optimal rhamnose concentration in Lemo21(DE3). **Expression of the gene encoding secretory BL1 was induced with IPTG in Lemo21(DE3) in the presence of 500 μM of rhamnose (see Figure 5). 4 h after induction a whole cell lysate was prepared and the periplasmic fraction isolated. **A **Nitrocellulose membranes containing increasing amounts of β-galactosidase were incubated with the whole cell lysate (top panel) and whole cell lysate that had been incubated with β-mercaptoethanol (middle panel). Binding of BL1 to the β-galactosidase spotted on the nitrocellulose membranes was detected using an α-His antibody recognizing the C-terminal His-tag of BL1. A nitrocellulose membrane containing spots with increasing amounts of BSA and incubated with the same lysate used in the top panel was included as a control (bottom panel). **B **The periplasmic fraction was isolated as described in Methods. SEC was used to analyze the BL1 that was isolated from the periplasmic fraction by means of IMAC. Indicated fractions (inset) from the SEC were analyzed by SDS-PAGE followed by Coomassie staining (top panel inset) or immuno-blotting using an α-His antibody (middle panel inset). The fractions representing the indicated peak were pooled and the BL1 was tested for binding to β-galactosidase (bottom panel inset). β-galactosidase concentrations correspond to the setup described in **A. **The bottom panel of the inset shows only the first 4 concentrations.

To address if the optimization of the production of processed BL1 results in properly folded protein, we examined the binding ability to its substrate, β-galactosidase [25]. A whole cell lysate from Lemo21(DE3) cells producing BL1 at a rhamnose concentration of 500 μM was prepared. At this rhamnose concentration, only the processed form of BL1 could be detected (Figure 5B and Additional file 1: Figure S1 and Figure S2). The substrate of BL1, β-galactosidase, was spotted in decreasing concentrations on a nitrocellulose membrane, which was then incubated with the whole cell lysate. BL1 that bound to β-galactosidase was detected using an α-His antibody. Bovine serum albumine (BSA) was used as a negative control. Using this set-up, we showed that at least part of the BL1 produced at the optimal rhamnose concentration was capable of binding to its substrate (Figure 6A). Treatment of whole cell lysate with the reducing agent β-mercaptoethanol prior to the incubation prevented binding of BL1 to β-galactosidase (Figure 6A), indicating the presence of structural disulfide bonds. Mass spectrometry analysis was used to further explore this observation. To this end, mature BL1 isolated from whole cells was treated with iodacetamide only or a reducing agent and iodactetamide (Additional file 1: Figure S3). Iodacetamide alkylates free cysteins and consequently the mass of the protein increases. Treatment with iodacetamide only did not increase the mass of mature BL1, whereas treatment with iodacetamide and a reductant led to a clear increase in mass. At the same time, isolated BL1 lost its ability to bind to β-galactosidase upon reduction/alkylation (Additional file 1: Figure S4). Taken together, these data show that disulfide bonds are indeed formed between the free cysteines of BL1 upon production in the periplasm and that they are important for the activity of the protein.

As a final step in the characterization of BL1 produced in the periplasm of Lemo21(DE3) cells cultured at 500 μM rhamnose, the protein was purified from the periplasmic fraction using immobilized metal affinity chromatography (IMAC). To assess the homogeneity of this purified material it was analyzed by means of size exclusion chromatography (SEC) (Figure 6B). The shape of the SEC trace was symmetric, indicating that the BL1 isolated from the periplasm was homogenous. Finally, the BL1 present in the fractions representing the symmetric peak was pooled and was shown to be active using the aforementioned β-galactosidase binding assay (Figure 6B, bottom panel inset).

Taken together, optimizing the expression level of the gene encoding secretory BL1 results in processed and properly folded protein in the periplasm.

## Discussion

There are two main reasons to produce heterologous proteins in the periplasm rather than in the cytoplasm of *E. coli*. Firstly, the isolation of proteins from the periplasm is usually easier than the isolation of proteins from total cell lysates, since the periplasm represents a less complex protein mixture than the cytoplasm [2]. Secondly, the Dsb-system in the periplasm can catalyze the formation of disulfide bonds, whereas the reducing cytoplasm prevents disulfide bond formation [4,5]. To produce heterologous proteins in the periplasm, they are equipped with an N-terminal signal sequence so that they can traverse the cytoplasmic membrane *via* the Sec-translocon. Unfortunately, the production of heterologous secretory proteins in *E. coli* often has a severe negative effect on the formation of biomass and the yields of processed and properly folded material in the periplasm are frequently low [6]. To improve periplasmic yields of heterologous proteins, it is necessary to identify the bottlenecks hampering their production.

Here, we have used Lemo21(DE3) to identify what hampers the production of heterologous secretory proteins in *E. coli*. Two heterologous secretory proteins, SFGFP and the scFv BL1, were produced in Lemo21(DE3) following varying gene expression levels. A derivative of the *E. coli* DsbA signal sequence, which funnels proteins into the co-translational SRP-targeting pathway and is widely used to produce heterologous secretory proteins in *E. coli*, was used to guide the two proteins to the Sec-translocon. Modulating the expression levels of the genes encoding the two heterologous secretory proteins not only had clear effects on the fitness of the cells producing SFGFP and BL1, but also on the levels of SFGFP and BL1 in the periplasm. Notably, our data show that only a narrow window of expression levels of the genes encoding the targets results in optimal protein yields in the periplasm. Sub-optimal conditions, i.e., too high gene expression levels, led to impaired growth and low protein yields and too low gene expression levels led to very low periplasmic protein yields. The protein misfolding/aggregation problem in the cytoplasm at high gene expression levels indicates that heterologous secretory proteins accumulate in the cytoplasm as a result of saturating the Sec-translocon capacity. This was corroborated by the impaired translocation of the endogenous secretory proteins OmpA and MalE. The cytoplasmic accumulation of endogenous secretory proteins, whose signal sequences are aggregation-prone, and membrane proteins will lead to the misfolding/aggregation of proteins in the cytoplasm [19]. Both OmpA and MalE are targeted to the Sec-translocon in a SecB-dependent rather than in an SRP-dependent fashion [33]. This indicates that saturation of the SRP-targeting pathway is not a bottleneck. Notably, the consequences of the production of membrane proteins in *E. coli* at too high gene expression levels resemble exactly the consequences of the production of SFGFP and the scFv BL1 equipped with a DsbA-derived signal sequence at too high gene expression levels [20,21]. This strongly supports the notion that saturation of the Sec-translocon capacity is the main bottleneck hampering the production of the two model secretory proteins used in this study when the gene expression levels are too high.

Already during the mid 90’s of last century some interesting observations as to the production of secretory proteins in *E. coli* were made that have been waiting for an explanation ever since. It was shown that levels of periplasmic PhoA in *E. coli* could be markedly enhanced when *phoA* transcript levels were reduced [35]. In a subsequent study it was shown that the ability of cells to secrete proteins into the periplasm was impaired upon overexpression of *phoA*[36]. Furthermore, it was shown that the production of secretory proteins in the periplasm of *E. coli* could be improved by random alteration of the translational initiation region of a signal sequence of a secretory protein [37]. This resulted in varied translational strengths, which had a great impact on protein yields in the periplasm. Our work suggests that these observations can likely be explained by the relief of the saturation of the Sec-translocon capacity upon moderation of the target protein production levels.

We show in this study that the Sec-translocon capacity can be a major bottleneck hampering the production of proteins in the periplasm. It should be kept in mind though that there may be additional bottlenecks*.* For some proteins the co-expression of genes encoding periplasmic chaperones and components of the Dsb-system can improve their levels in the periplasm [6]. This indicates that chaperone capacity in the periplasm can also be limiting for the production of proteins in this compartment. However, it is also possible that co-expression of genes encoding periplasmic factors assisting protein folding helps in clearing the Sec-translocon, thereby increasing Sec-translocon capacity [38].

## Conclusions

The Sec-translocon capacity can be a major bottleneck hampering the production of proteins in the periplasm of *E. coli*. Harmonizing the expression levels of the gene encoding the heterologous secretory protein with the Sec-translocon capacity alleviates this bottleneck. Optimal yields can only be achieved within a narrow window of gene expression levels. Importantly, our study shows that mechanistic insight into the production of proteins in the periplasm is key to optimizing yields in this compartment.

## Methods

### Strains and plasmids

To modulate the expression levels of the genes encoding secretory SFGFP [23] and BL1 [24,25] in *E. coli,* the Lemo21(DE3) strain was used. Lemo21(DE3) is a BL21(DE3) derivative, harboring a pACYC-derived vector containing the gene encoding the T7 lysozyme under the control of the rhamnose promoter (Figure 2). Notably, the T7 lysozyme K128Y variant that has no amidase activity but retains full inhibition of T7 RNA polymerase was used [39]. The BL21(DE3) strain was used as a reference. The proteins used in that study were expressed from a pET28a+ derived vector as described before [21]. The sequence encoding SFGFP was obtained from E.L. Snapp and the gene was synthesized by GeneArt [22]. The genes encoding SFGFP and BL1 were fused to the genetic information encoding a DsbA derived signal sequence (atg tta aga tcc atg aaa aag att tgg ctg gcg ctg gct ggt tta gtt tta gcg ttt agc gca tcg gcg) at the 5^′^ end. BL1 is equipped with a C-terminal His-tag. For cytoplasmic expression of SFGFP, the gene encoding only SFGFP was used. Lemo21(DE3) transformed with a pET28a+ derived, “empty” expression vector served as a negative control.

### Culture media and expression conditions

Cells were grown aerobically at 30°C and 200 rpm, in Lysogeny broth (LB) medium (Difco) supplemented with 50 μg/ml kanamycin and 30 μg/ml chloramphenicol (Lemo21(DE3) only). Lemo21(DE3) was grown in the absence and presence of increasing concentrations of rhamnose as indicated. At an A_600_ of ~0.4 protein expression was induced by adding 400 μM IPTG for 4 h. Growth was monitored by measuring the A_600_ with an UV-1601 spectrophotometer (Shimadzu). Standard deviations shown in figures of culturing experiments are based on at least three biologically independent experiments.

### Whole cell fluorescence measurements and flow cytometry

Expression of secretory SFGFP was monitored using whole-cell fluorescence essentially as described before [40]. Due to the intense fluorescence of cells expressing SFGFP a volume of 100 μl was used for the whole-cell fluorescence measurements. For displaying purposes, the highest expression levels per volume (fluorescence unit/ml) was set to 100%. Standard deviations are based on a minimum of three biologically independent experiments.

Cell size and granularity were analyzed by flow cytometry using a FACSCalibur instrument (BD Biosciences) essentially as described before [20,33,41,42]. FM4-64 membrane staining was used to discriminate between cells and background signal. The FlowJo software (Treestar) was used for raw data analysis/processing.

### SDS-PAGE and immuno-blotting

Whole cell lysates (0.05 A_600_ units) were analyzed by standard SDS-PAGE using standard polyacrylamide gels followed by immuno-blotting as described before [20]. Secretory BL1 was detected using an HRP-conjugated α-His antibody (ThermoFisher) recognizing the C-terminal His-tag. T7 lysozyme, IbpB, OmpA and MalE levels were monitored using respective antisera from our sera collection, followed by incubation with a secondary HRP-conjugatedgoat-α-rabbit antibody (Bio-Rad). Proteins were visualized using the ECL-system (GE Healthcare) according to the instructions of the manufacturer and a Fuji LAS-1000 charge coupled device (CCD) camera.

### Fluorescence microscopy

Prior to microscopy, cells were fixed using cross-linking reagents. Cells corresponding to 1 A_600_ unit were harvested (4000 × *g*, 2 min) and resuspended in 1 ml phosphate buffered saline (PBS) pH 7.4. Subsequently, 1 ml fixing solution (5.6% Formaldehyde, 0.08% Glutaraldehyde in PBS) was added and cells were incubated for 15 min at room temperature (RT). Subsequently, cells were washed three times with PBS and resuspended in 100 μl PBS. 1 μl of the cell suspension was mounted on a glass slide. Fluorescence images of cells expressing secretory SFGFP were obtained using a light scanning microscope (LSM 700) set-up (Zeiss). The resulting images were processed with the AxioVision 4.5 software (Zeiss).

### Preparation of whole cell lysate and BL1 activity assay

The proper folding of BL1 was assayed by the recognition of its substrate, *E. coli* β-galactosidase, using a dot-blot assay and whole cell lysate. Whole cell lysate was obtained as follows: 35 ml of a Lemo21(DE3) culture expressing secretory BL1 in the presence of 500 μM rhamnose were harvested by centrifugation (8000 × *g*, 20 min, 4°C) and subsequently resuspended in 1x PBS supplemented with 0.5 mg/ml PefablocSC, 25 μg/ml DNase and 0.05 mg/ml lysozyme to a final concentration of 10 A_600_ units/ml. Cell lysis was performed by passing the cells five times through an Emulsiflex-C3 (Avestin), at 10.000-15.000 psi. The lysate was cleared of unbroken cells by centrifugation (8000 × *g*, 20 min, 4°C).

For the activity assay, 2 μl of a serial dilution of β-galactosidase (5 mg/ml, 2.5 mg/ml, 1.25 mg/ml, 0.625 mg/ml, 0.31 mg/ml, 0.15 mg/ml) were spotted directly onto a nitrocelullose membrane (Millipore) using a BIO-DOT device (Bio-Rad). As a negative control, the same amounts of BSA were spotted on a separate membrane. Non-specific binding sites were blocked by incubating the membrane with a solution of tris buffered saline containing 0.05% Tween 20 (TBS-T) with 5% milk for 1 h at RT. Membranes were washed for three times 15 min with TBS-T and subsequently incubated for 1 h at RT with the whole cell lysate. As a negative control, membranes containing β-galactosidase were treated with 5-6 ml cell lysates incubated with β-mercaptoethanol (140 μl/A_600_ unit). Binding of BL1 was visualized using an HRP-conjugated α-His antibody (Pierce), the ECL-system (GE Healthcare) and a Fuji LAS-1000 CCD camera.

### Isolation of periplasmic fraction and purification of BL1

Cells from 4x 1 l cultures were harvested by centrifugation (8000 × g, 30 min, 4°C) and the cell pellet was snap-frozen in liquid nitrogen. All subsequent steps were carried out on ice or at 4°C. The pellet was resuspended under gentle agitation in 1 ml ice-cold periplasmic isolation buffer (500 mM sucrose, 100 mM Tris, 1 mM EDTA, pH 8) per 120 A_600_ units of cells, supplemented with 0.5 mg/ml PefablocSC. Spheroplast formation and release of the periplasmic fraction were facilitated by six cycles of 5 min incubation at 4°C followed by 10 sec of vortexing. Spheroplasts were removed by centrifugation (10.000 × *g*, 30 min) and the supernatant used for purification of BL1.

BL1 was isolated from the supernatant after the isolation of the periplasmic fraction using a combination of IMAC and SEC. Imidazole and MgCl_2_ concentrations of the supernatant were adjusted to 10 mM and 2 mM, respectively. 0.5 ml of Ni-beads (Qiagen) were added to 50 ml of the supernatant and the mixture was incubated at 4°C for 1 h on a rocking table. Ni-beads were concentrated (3000 × *g*, 10 min, 4°C) and loaded onto a gravity column. The column was washed with 5 column volumes of washing buffer (50 mM NaH_2_PO_4_, 300 mM NaCl, 20 mM imidazole pH 8). BL1 was eluted with 2,8 ml elution buffer (50 mM NaH_2_PO_4_, 300 mM NaCl, 250 mM imidazole pH 8) and six fractions were collected. Fractions containing BL1 (determined by immunoblotting using an α-His antibody) were pooled and diluted in 8 ml gel filtration buffer (150 mM NaCl dissolved in 1 × PBS, pH 7.4) The sample volume was reduced to 0.5 ml by usin*g* a vivaspin 20 concentrator (Satoriusstedim) and the sample was loaded onto a 24 ml Superdex 200 10/300 GL column (GE Healthcare Bio-Sciences, Uppsala, Sweden) using an ÄKTA Prime Plus purification system (GE Healthcare Bio-Sicence). 0.5 ml fractions of the flow-through were collected at a flow rate of 0.2 ml/min. The elution profile was monitored by using the Prime View software (GE Healthcare Bio-Science). Eluted fractions were analyzed by SDS-PAGE followed by coomassie staining and immuno-blotting. Fractions containing no detectable contaminants were pooled and analyzed with the above described BL1 activity assay using 2 ml of solution containing BL1 at a final concentration of 17.8 μg/ml.

## Abbreviations

Dsb: Disulfide bond formation; SRP: Signal recognition particle; SFGFP: Super folder green fluorescent protein; scFv: Single-chain variable antibody fragment; RNAP: RNA polymerase; IPTG: Isopropyl-β-D-thiogalactoside; IbpB: Inclusion body protein B; BSA: Bovine serum albumin; IMAC: Imobilized metal affinity chromatography; SEC: Size exclusion chromatography; LB: Lysogeny broth; CCD: Charge coupled device; PBS: Phosphate buffered saline; TBS-T: Tris buffered saline 0.05% Tween 20; RT: Room temperature; CM: Cytoplasmic membrane; Lep: Leader peptidase; OM: Outer membrane.

## Competing interests

The authors declare that they have no competing interests.

## Authors’ contributions

SS designed and carried out experiments, analyzed data and helped to write the manuscript. ER designed and carried out experiments and analyzed data. AJY designed and carried out experiments, and analyzed data. RAZ provided access to crucial infrastructure. JL designed experiments, analyzed data and helped to write the manuscript. JWdG designed experiments, analyzed data and helped to write the manuscript. All authors read and approved the final manuscript.

## Supplementary Material

Additional file 1: Figure S1 Subcellular fractionation of BL21(DE3) and Lemo21(DE3) cells producing secretory BL1. **Figure S2. **Mass spectrometry analysis of secretory BL1 produced in Lemo21(DE3). **Figure S3. **Mass spectrometry analysis of the redox state of the four cysteines present in mature BL1. **Figure S4. **Binding of mature, purified BL1 to β-galactosidase. **Comment **The production of secretory SFGFP is discussed in the light of recent publications. Click here for file
