# Supplementary material for: Optimizing heterologous protein production in the periplasm of E. coli by regulating gene expression levels
Source: Microb Cell Fact. 2013 Mar 12;12:24. doi: 10.1186/1475-2859-12-24 (PMC3605120; doi:10.1186/1475-2859-12-24)
Supplement: Additional file 1: Figure S1 — Subcellular fractionation of BL21(DE3) and Lemo21(DE3) cells producing secretory BL1. Figure S2. Mass spectrometry analysis of secretory BL1 produced in Lemo21(DE3). Figure S3. Mass spectrometry analysis of the redox state of the four cysteines present in mature BL1. Figure S4. Binding of mature, purified BL1 to β-galactosidase. Comment The production of secretory SFGFP is discussed in the light of recent publications. [file 1475-2859-12-24-S1.pdf]

Additional material

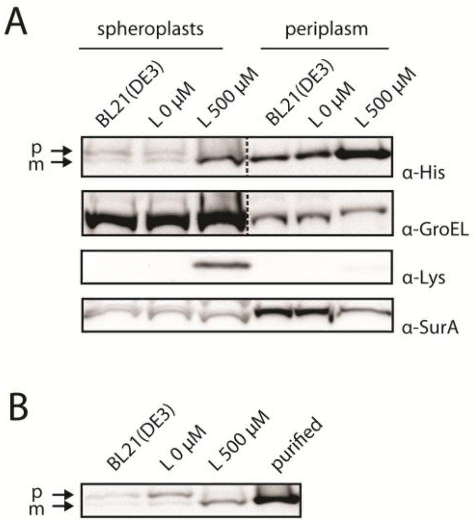

Figure S1 Subcellular fractionation of BL21(DE3) and Lemo21(DE3) cells producing secretory BL1. Secretory BL1 was expressed in BL21(DE3) and Lemo21(DE3) in the absence (L 0 µM) and presence of 500 µM (L 500 µM) rhamnose as described in the 'Methods' section. **A** Spheroplasts were generated and the periplasmic fraction isolated essentially as described in [1]. All steps were carried out on ice/at 4°C. Protein concentrations were determined using the BCA assay (Pierce). Sample volumes corresponding to 7.5 µg of total protein were analyzed using SDS-PAGE followed by immuno-blotting. BL1 was detected using an α-His antibody as described in the 'Method' section. The chaperone GroEL (as described by [2]) and T7 lysozyme (Lys) were used as cytoplasmic markers, The chaperone SurA was used as a periplasmic marker. Note that the lane with the molecular weight marker was excluded from the figure for the panels α-His and α-GroEL (indicated by the vertical, dashed line). **B** To confirm that the lower molecular weight form represents the mature form of BL1 whole cell lysates (corresponding to 7.5 µg of total protein) were analysed by SDS-PAGE/immunoblotting using 1 µg of purified, mature BL1 (purified) as a reference. BL1 was detected using an α-His antibody. The precursor (p) form and mature (m) form of the protein are indicated.

References

1. Martin CD, Rojas G, Mitchell JN, Vincent KJ, Wu J, McCafferty J, Schofield DJ: **A simple vector system to improve performance and utilisation of recombinant antibodies**. *BMC Biotechnol* 2006, **6**:46.
2. Fisher AC, DeLisa MP: **Laboratory evolution of fast-folding green fluorescent protein using secretory pathway quality control**. *PLoS One* 2008, **3**(6):e2351.

A

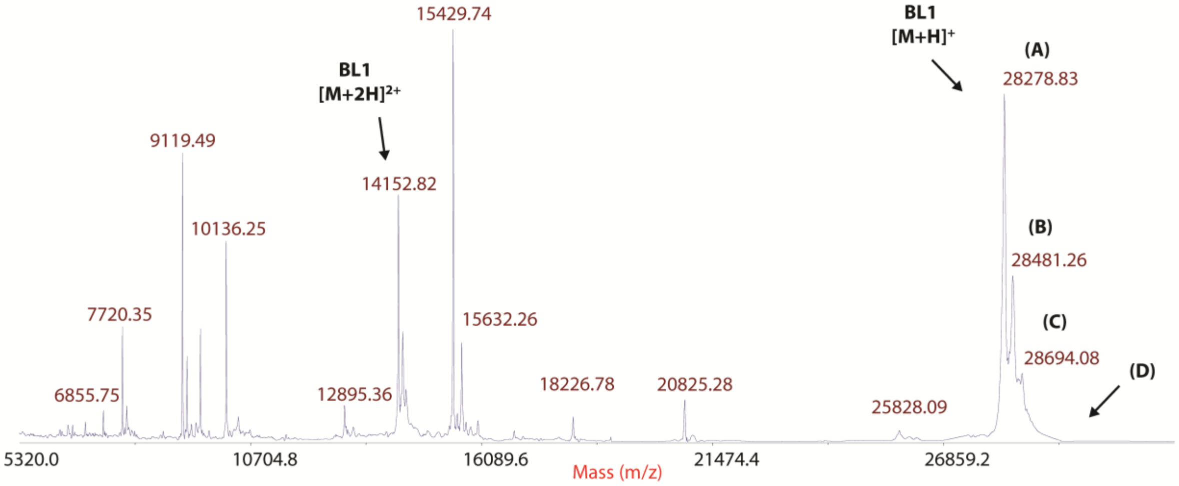

B

peak	mass [Da]	protein form	aminoacids	predicted average mass [Da]	deviation
(A)	28,280.1 ± 6.1	mature	26-297	28,325.2	-45.1 (0.16%)
(B)	28,479.1 ± 6.7	mature	24-297	28,527.5	-48.4 (0.17%)
(C)	28,692.6 ± 5.1	mature	22-297	28,685.7	+6.9 (0.02%)
(D)	not observed	precursor	1-297	30,968.2	N/A

C

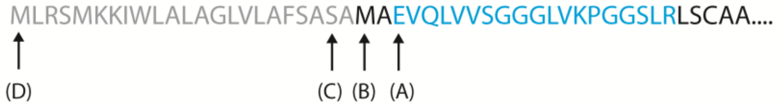

Figure S2 Mass spectrometry analysis of secretory BL1 produced in Lemo21(DE3). BL1 was isolated from Lemo21(DE3) cells cultured in the presence of 500 µM rhamnose and subsequently analyzed using mass spectrometry. **A** The purified protein was desalted using custom made microcolumns and eluted directly onto the MALDI target using sinapinic acid as matrix. The mass of the intact protein was determined using a Voyager De-PRO MALDI-TOF MS (PerSeptive Biosystems). Peaks corresponding to BL1 are indicated. **B** Detailed description of peaks corresponding to BL1. Masses represent the average of 3 measurements. Predicted masses were determined using the Expasy Peptide Mass Calculator (found at http://web.expasy.org). Deviations between predicted and measured masses in Dalton and % are indicated. **C** N-terminal amino acid sequence of secretory BL1 representing the DsbA-derived signal sequence (grey) and the N-terminal part of the mature BL1. The start sites of the processed forms of secretory BL1 as identified by MS (A),(B),(C) and the precursor form of BL1 (D) are indicated by arrows. For unambiguous identification of BL1 the protein was digested by trypsin and analyzed on a Q Exactive MS (Thermo Scientific) to generate MS/MS. The identified peptide sequence is displayed in blue and corroborates the cleavage at (A). Apparently the signal peptidase can cleave secretory BL1 at different positions, which is not unusual.

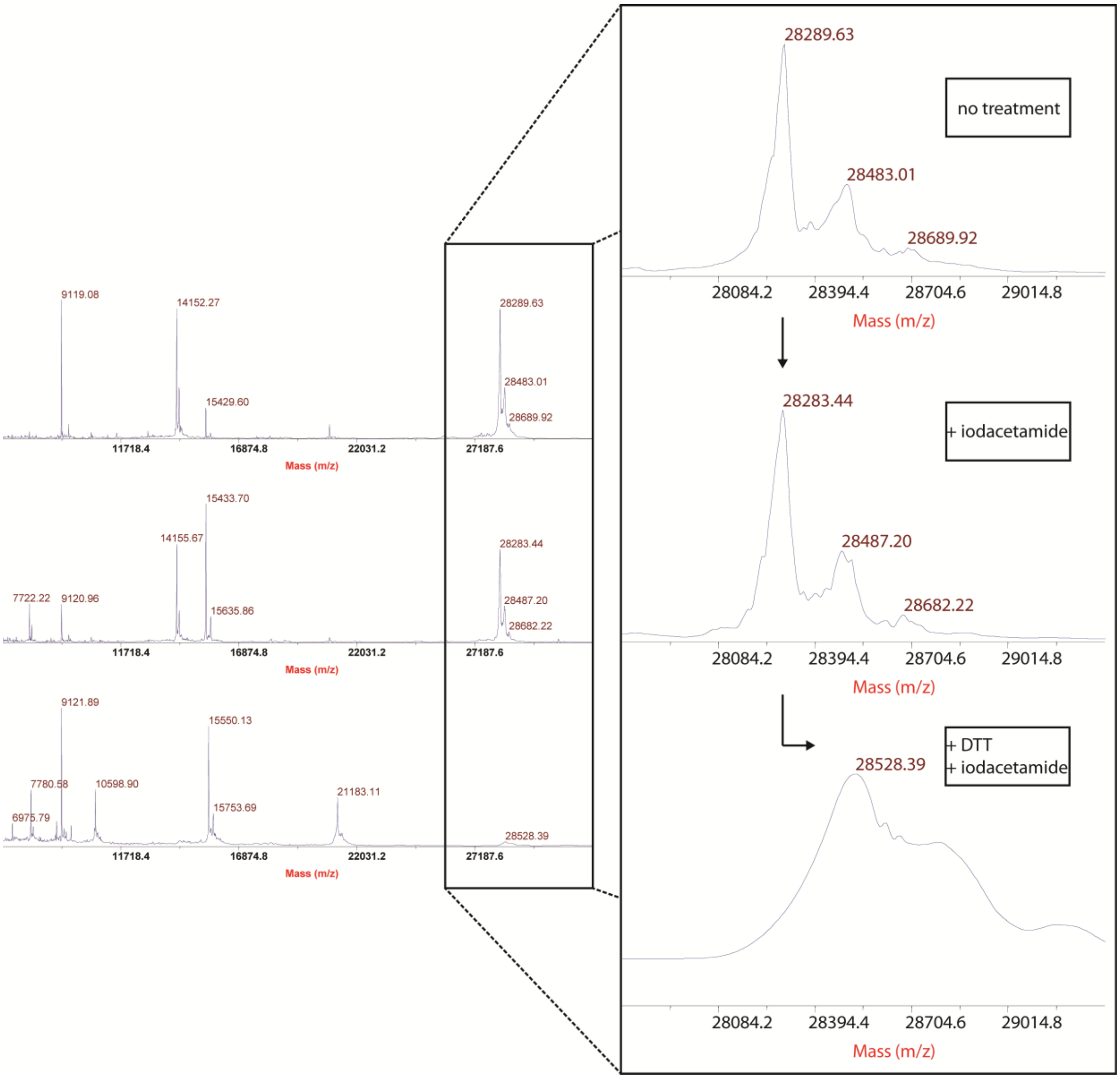

Figure S3 Mass spectrometry analysis of the redox state of the four cysteines present in mature BL1. BL1 was isolated from Lemo21(DE3) cells cultured in the presence of 500 µM rhamnose (see Figure S2). BL1 was treated as described below and subsequently analyzed using a Voyager De-PRO MALDI-TOF MS. To determine if any of the cysteines were involved in disulfide bonds the intact mass of BL1 was measured without treatment, alkylation (iodoacetamide) and reduction (DTT) and alkylation together. Iodoacetamide alkylates free cysteines. Comparing the top and the bottom spectra shows that reduced and alkylated BL1 increase in mass by 239 Da, which is consistent with alkylation of all four cysteines. The lack of a shift in the middle spectrum indicates that all four cysteines present in mature BL1 are involved in disulfide bonds.

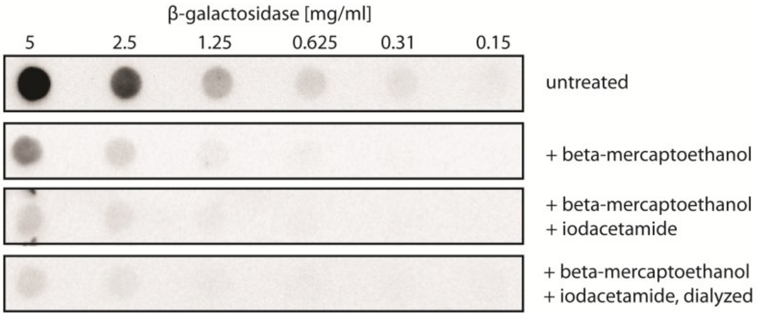

Figure S4 Binding of mature, purified BL1 to β-galactosidase. The ability of BL1 isolated from whole cell lysates (compare Figure S1) to bind to its substrate β-galactosidase was determined using the dot-blot approach as described in the 'Methods' section using a BL1 solution with a final concentration of 0.01 µg BL1/µl. The BL1 solutions used for the dot-blot assays were treated as follows: incubation without any reagent as a control (lane 1); + β-mercaptoethanol: incubated with 210 µl β-mercaptoethanol/sample (2 ml) (lanes 2-4); + iodacetamide: iodacetamide added to saturation after β-mercaptoethanol treatment followed by an adjustment of the final volume to 3 ml (lanes 3, 4); dialyzed: iodacetamide/β-mercaptoethanol treated sample dialyzed using a ThermoFisher SnakeSkin dialysis membrane with a cutoff of 7 kDa.

Comment (see page 5 in the manuscript)

It has been suggested that under some conditions secretory SFGFP can fold in the cytoplasm [1]. However, fluorescence microscopy revealed a halo of fluorescence in Lemo21(DE3) cells producing secretory SFGFP (Figure 3C). It has been shown that *E. coli* cells producing secretory SFGFP have a similar halo of fluorescence [2, 3]. Periplasmic fractionations showed that the halo of fluorescence in these cells indeed represents processed periplasmically localized SFGFP [2, 3]. Although we could not fractionate cells producing secretory SFGFP we feel, in spite of the evidence that secretory SFGFP may be able to fold in the cytoplasm, confident that the halos of fluorescence observed in Lemo21(DE3) cells producing secretory SFGFP represent periplasmically localized SFGFP.

References

1. Fisher AC, DeLisa MP: **Laboratory evolution of fast-folding green fluorescent protein using secretory pathway quality control**. *PLoS One* 2008, **3**(6):e2351.
2. Aronson DE, Costantini LM, Snapp EL: **Superfolder GFP is fluorescent in oxidizing environments when targeted via the Sec translocon**. *Traffic* 2011, **12**(5):543-548.
3. Dinh T, Bernhardt TG: **Using superfolder green fluorescent protein for periplasmic protein localization studies**. *J Bacteriol* 2011, **193**(18):4984-4987.
